# Circulating microRNAs have a sex-specific association with metabolic syndrome

**DOI:** 10.1186/1423-0127-20-72

**Published:** 2013-10-04

**Authors:** Yu-Ting Wang, Pei-Chien Tsai, Yi-Chu Liao, Chung-Y Hsu, Suh-Hang Hank Juo

**Affiliations:** 1 Department of Genome Medicine, Kaohsiung Medical University, Kaohsiung 807, Taiwan; 2 Department of Medical Research, Kaohsiung Medical University Hospital, Kaohsiung 807, Taiwan; 3 Department of Internal Medicine, National Taiwan University Hospital Yun-Lin Branch, Douliu 640, Taiwan; 4 Section of Neurology, Taichung Veterans General Hospital, Taichung 407, Taiwan; 5 Department of Neurology, School of Medicine, National Yang-Ming University, Taipei 112, Taiwan; 6 Department of Neurology, Kaohsiung Medical University Hospital, Kaohsiung 807, Taiwan; 7 Institute of Clinical Medical Science, China Medical University, Taichung 404, Taiwan; 8 Department of Neurology, China Medical University Hospital, Taichung 404, Taiwan

**Keywords:** Obesity, Gender disparity, let-7 g, miR-221

## Abstract

**Background:**

The microRNAs let-7 g and miR-221 have been demonstrated to be related to the glucose metabolism. This study assessed the serum levels of these two microRNAs in subjects with and without metabolic syndrome (MetS).

**Results:**

The serum microRNA levels were detected in 102 subjects aged 40 to 80 years who were recruited from the general population. The status of MetS was defined by the Adult Treatment Panel III (ATP III) criteria modified for Asians. Subjects with histories of cardiovascular diseases or who were receiving treatment with hypoglycemic or lipid-lowering agents were excluded. The levels of both circulating microRNAs (let-7 g and miR-221) were higher in subjects with MetS (p = 0.004 and p = 0.01, respectively). The sex-specific analysis showed that the difference was more prominent in women (for both miRNAs, p < 0.05 in women and p > 0.1 in men). In the female subjects, increased expression of both microRNAs was associated with an increased number of MetS risk components (p = 0.002 for let-7 g and p = 0.022 for miR-221). Moreover, the elevation of serum let-7 g was significantly associated with a low level of high-density lipoprotein cholesterol (p = 0.022) and high blood pressure (p = 0.023). In contrast, the miR-221 level was not associated with any individual MetS risk component.

**Conclusions:**

The circulating levels of let-7 g and miR-221 displayed a female-specific elevation in individuals with metabolic syndrome.

## Background

Metabolic syndrome (MetS) has become a global health issue due to its contribution to an increased risk of type 2 diabetes, cardiovascular (CV) complications, and mortality. MetS features a clinical phenotype of abdominal adiposity, insulin resistance, hypertension, dyslipidemia, inflammation, and a prothrombotic state. The associations between MetS and chronic inflammation [1], insulin resistance [2], or incident CV events [3] have been presented differently between men and women. Furthermore, a recent study demonstrated that gender is a significant effect modifier for MetS-related CV deaths, non-CV deaths, and total mortality [4].

MicroRNAs (miRNAs), which are endogenous non-coding small RNAs, are involved in the post-transcriptional regulation of gene expression through the suppression of mRNA translation or the degradation of mRNA. More than 60% of the human protein-coding genes are expected to have miRNA target sites in their 3’-untranslated region (3’-UTR); therefore, miRNAs are considered to likely be involved in the pathogenesis of diseases [5-8]. An ample of evidence indicates that miRNAs can be detected in the circulation [9,10]. After being released into the extracellular fluids, miRNAs play roles in cell-to-cell communication within the same tissue or between the same and different cell types in remote tissues by the methods of endocytosis-like internalization or receptor-ligand interactions [11].

One recent study compared subjects with MetS to individuals manifesting only one of metabolic disorders [type 2 diabetes, hypercholesterolemia, or hypertension] showed that blood miR-197, miR-23, and miR-509-5p positively correlated with body mass index, and elevated circulating miR-130a and miR-195 were associated with high blood pressure [12]. Using *in silico* analysis, the authors predicted that the target genes of these 5 miRNAs may involve in the pathways related to shingolipid and fatty acid metabolisms and the vascular signaling. Metabolic syndrome is a diseased condition which affects both adipose tissue and vascular walls. Extracellular miRNAs may mediate the intercellular communication. The adipocyte-derived microvesicles contained not only bioactive proteins but also miRNAs, including let-7b and miR-103, 143, 146b, 148, 155, and 221 [13]. Of these, let-7b, miR-143, and 221 have been demonstrated to regulate both atherogenic and adipogenic processes [13]. Heneghan et al. compared the expression profiles of circulating miRNAs between morbidly obese patients and non-obese individuals and found miR-132 and miR-17-5p were significantly decreased in obese subjects [14]. The level of miR-17-5p inversely correlated with body mass index and it may regulate adipocyte differentiation by targeting Rb2/p130. In the same article, the data did not confirm differences in the levels of circulating miR-34a, 99a, 122, 143, 145, and 195 between subjects in the two groups. However, the authors did not make a mention of let-7 or miR-221.

Previous publications have indicated that the let-7 family plays an important role in glucose metabolism. Using *Cre*-inducible let-7-transgenic mice, Frost and Olson [15] reported that the global overexpression of let-7 resulted in glucose intolerance and decreased insulin secretion from the pancreas. The knockdown of let-7 can prevent and treat obesity-induced glucose intolerance by restoring the expression of the insulin receptor and insulin receptor substrate 2 in the muscle and liver of mice fed a high-fat diet [15]. Zhu et al. also found that Lin28a/b and let-7 can coordinately regulate the glucose metabolism through the activation and suppression of the insulin-PI3K-mTOR signaling pathway, respectively [16]. *In vitro* studies have shown that high glucose (HG) upregulates miR-221 expression in human umbilical vein endothelial cells and results in impaired endothelial cell migration through the suppression of the c-kit gene [17]. A reduction of the miR-221 levels through its anti-sense oligonucleotide could restore the production of the c-kit protein and weaken the HG-induced inhibition of cell migration. The levels of miR-221 are increased in adipocytes derived both from mice under a high-fat diet [18] and obese human subjects [19]. In addition, miR-221 can be upregulated in 3 T3-L1 adipocytes treated with tumor necrosis factor-alpha [20].

In the present study, we have found the association of two circulating microRNAs (let-7 g and miR-221) with metabolic syndrome in human subjects, more prominent in women. The over-expression of the two microRNAs in the circulation may have implications for MetS related CV complications.

## Methods

### Study population and definition of metabolic syndrome

The subjects in this study (aged between 40 and 80 years) were enrolled from the general population who had volunteered to participate in ongoing genetic studies related to cardiovascular diseases and metabolic syndrome at the Kaohsiung Medical University Hospital between Jan 2010 and Sep 2011. All of the participants provided written informed consent. To reduce the effect from existing major cardiovascular diseases, subjects who self-reported coronary artery disease, myocardial infarction, stroke, or peripheral arterial occlusive disease were excluded from the study. In addition, subjects who were taking medication for hyperglycemia or hyperlipidemia were also excluded. At the study entry, 122 subjects were enrolled but 20 were excluded due to the presence of hemolysis. Metabolic syndrome was diagnosed in patients who exhibited three or more of the following symptoms, which are defined by the Adult Treatment Panel (ATP) III criteria modified for Asians: 1) waist circumstance greater than 90 cm in men and greater than 80 cm in women, 2) systolic blood pressure greater than 130 mmHg, diastolic blood pressure greater than 85 mmHg, or previously diagnosed hypertension with treatment, 3) fasting glucose level greater than 100 mg/dl or previously diagnosed diabetes mellitus with treatment, 4) fasting triglycerides greater than 150 mg/dl, and 5) high-density lipoprotein (HDL) cholesterol less than 40 mg/dl in men and less than 50 mg/dl in women. The classification of metabolic syndrome from 0–5 was based on the number of abnormal metabolic components defined by the above criteria. A subject who reported never having smoked was defined as a never smoker. A subject who had previously smoked in his/her lifetime was defined as a smoker. Because the median age of natural menopause in Chinese women is 50 years [21,22], we further separated the women by this age cutoff for an exploratory analysis.

### Serum preparation and hemoglobin measurement for hemolysis

Within 1 h after sample collection, the fasting venous blood was centrifuged at 3,000 g and 4°C for 10 min. The serum was stored immediately at -80°C until further use. To determine the degree of hemolysis in the samples, 200 μl of sodium carbonate solution (0.01% Na_2_CO_3_) was added to 20 μl of each serum sample (10:1 dilution). After vortex mixing and incubating at room temperature for 1 h, the free oxy-hemoglobin in the serum was measured using a spectrophotometer at an absorbance wavelength of 415 nm (A_415_) [23]. Higher levels of the absorbance peak indicated a higher degree of hemolysis in the serum. The cutoff A_415_ value of 0.2 was used to distinguish the hemolyzed from the non-hemolyzed samples [24].

### Isolation of total RNA and miRNA from serum

The serum total RNA, including miRNAs, was extracted using the MasterPure™ Complete DNA and RNA Purification Kit (Epicentre, an Illumina company, USA) according to the manufacturer’s instructions. After effective denaturation of the proteins in serum during RNA isolation, we added 1 μl of 5 nM synthetic, non-human miRNA (syn-cel-lin-4) into 50 μl of each serum sample as an internal control for the normalization of the real-time PCR results. The final RNA products were quantified by absorbance measurements at 260 nm (A_260_) and 280 nm (A_280_). The A_260_/A_280_ values were higher than 1.6 for all of the samples.

### Quantification of miRNAs: real-time quantitative PCR

The mature miRNAs were measured using TaqMan-based qRT-PCR methods according to the manufacturer’s instructions (Applied Biosystems, Foster City, CA, USA). The reverse transcription reactions were performed in scaled-down reaction volumes (8 μl) under the following conditions: 16°C for 30 min, 42°C for 30 min, and 85°C for 5 min. The real-time PCR reactions were performed in duplicate in scaled-down reaction volumes (10 μl) on an ABI Prism 7900 Sequence Detection System under the following conditions: 95°C for 10 min, followed by 40 cycles at 95°C for 15 sec and 60°C for 1 min. The data were analyzed using the SDS Software version 2.4 (Applied Biosystems, Inc.), and a miRNA with a Ct value greater than 36 was considered undetectable. The analysis across the samples was conducted using a median normalization method with a spiked-in synthetic *C*. *elegans* miRNA (syn-cel-lin-4) as the control [10]. The normalization factors were obtained after subtracting the measured Ct of syn-cel-lin-4 for each sample of interest from the median value of all of the samples. Then, the normalization factor was added to the raw Ct of every candidate miRNA in the same sample. Finally, the adjusted data (Ct’) was treated as the median-normalized Ct value. The expression level of a circulating miRNA was calculated as 2^-Ct’^ with a logarithmic transformation.

### Statistical analysis

JMP (version 8.0) and PASW Statistics (version 18) were used for all statistical analyses. To evaluate the baseline characteristics and the risk components of MetS, Chi-square analyses and t-test were applied for the categorical and continuous variables, respectively. Comparisons of the levels of circulating miRNAs between the two groups were conducted by the non-parametric Mann–Whitney *U* tests because the data were not normally distributed. The relationship between the number of MetS components and the expression of both miRNAs was tested through the Jonckheere-Terpstra test. Correlation coefficients were used to examine the relationships between the value of each MetS risk component and the expression level of a miRNA. A difference with p < 0.05 was considered statistically significant.

## Results

### Clinical characteristics of the study population

Because hemolysis can increase the serum microRNA levels due to red blood cells (RBC)-derived microRNAs [24], we excluded 20 serum samples that exceeded the hemolysis cutoff value (see Method section for details). Therefore, 102 samples were subjected to further analysis. The demographic characteristics and the status of MetS in these 102 subjects are shown in Table 1. There were 31 MetS subjects and 71 non-MetS. In general, the distributions of sex, age, prevalence of diabetes, and smoking status were similar between the groups with or without MetS. The characteristics between men and women in respect of their MetS status are also shown in Table 1. The prevalence of each risk component between genders according to the sum of abnormal components of the MetS is shown in Table 2. Of the five components of MetS, large waist circumstance was the most common risk factor in both genders, followed by high blood pressure. However, a higher prevalence of decreased HDL cholesterol was noted in women (28%) than in men (13.5%), whereas fasting hyperglycemia was more common among men (36.5%) than women (24%).

**Table 1:** Characteristics of the subjects with and without metabolic syndrome

	**All (n = 102)**			**Men (n = 52)**			**Women (n = 50)**		
	**without MS**	**with MS**	***p *value**	**without MS**	**with MS**	***p *value**	**without MS**	**with MS**	***p *value**
Total number	71	31		36 (69.2%)	16 (30.8%)		35 (70.0%)	15 (30.0%)	
Age (yr)	55.5 ± 7.8	56.5 ± 8.5	> 0.1	56 ± 9	55 ± 8	> 0.1	55 ± 6	58 ± 9	> 0.1
Smoker, n (%)	10 (14.1%)	4 (12.9%)	> 0.1	10 (27.8%)	4 (25.0%)	> 0.1	0 (0.0%)	0 (0.0%)	1.000
Hypertension, n (%)	14 (19.7%)	19 (61.3%)	< 0.0001	8 (22.2%)	11 (68.8%)	0.001	6 (17.1%)	10 (53.3%)	0.009
Anti-HTN drugs, n (%)	5 (7.0%)	6 (19.4%)	0.07	2 (5.6%)	4 (25%)	0.06	3 (8.6%)	2 (13.3%)	> 0.1
Systolic BP (mmHg)	114 ± 13	129 ± 13	< 0.0001	116 ± 12	128 ± 14	0.004	111 ± 13	131 ± 13	< 0.0001
Diastolic BP (mmHg)	71 ± 10	79 ± 9	0.0003	72 ± 10	79 ± 9	0.020	70 ± 10	79 ± 9	0.007
Diabetes, n (%)	2 (2.8%)	2 (6.5%)	> 0.1	1 (2.8%)	1 (6.3%)	> 0.1	1 (2.9%)	1 (6.7%)	> 0.1
Fasting sugar (mg/dl)	94 ± 18	107 ± 19	0.0013	93 ± 9	107 ± 15	0.001	91 ± 7	107 ± 23	0.0006
Body weight (kg)	63.0 ± 11.2	70.5 ± 12.6	0.0033	69.0 ± 10.7	77.6 ± 8.8	0.008	56.7 ± 7.8	63.0 ± 12.0	0.031
Body mass index (kg/m^2^)	24.1 ± 3.3	26.6 ± 3.6	0.0008	24.3 ± 3.4	26.9 ± 2.4	0.007	23.8 ± 3.2	26.2 ± 4.5	0.042
Waist circumference (cm)	82.7 ± 9.2	90.7 ± 7.5	< 0.0001	84.9 ± 8.6	92.3 ± 6.1	0.001	80.5 ± 9.5	89.0 ± 8.7	0.005
Waist-hip ratio	0.87 ± 0.07	0.92 ± 0.06	0.0019	0.89 ± 0.05	0.93 ± 0.04	0.006	0.85 ± 0.08	0.90 ± 0.07	0.046
Triglyceride (mg/dl)	95 ± 39	164 ± 93	0.0003	98 ± 45	166 ± 95	0.013	92 ± 32	163 ± 95	0.013
HDL cholesterol (mg/dl)	60 ± 14	50 ± 14	0.0008	55 ± 11	45 ± 10	0.003	66 ± 14	56 ± 15	0.022
Total cholesterol (mg/dl)	209 ± 39	216 ± 35	> 0.1	202 ± 38	209 ± 37	> 0.1	217 ± 38	224 ± 33	> 0.1

The data are expressed as a number (percentage) or mean ± SD. *MS*, metabolic syndrome; *HTN*, hypertension, *BP*, blood pressure, *HDL*, high density lipoprotein.

**Table 2:** Prevalence of individual metabolic syndrome component in men and women according to the number of abnormal components

	**Men**				**Women**			
	**Number of abnormal metabolic components**				**Number of abnormal metabolic components**			
	**0**-**5**	**0**-**2**	**3**	**4**-**5**	**0**-**5**	**0**-**2**	**3**	**4**-**5**
	**(All)**	**(without MS)**	**(with MS)**	**(with MS)**	**(All)**	**(without MS)**	**(with MS)**	**(with MS)**
	**(n = 52)**	**(n = 36)**	**(n = 12)**	**(n = 4)**	**(n = 50)**	**(n = 35)**	**(n = 10)**	**(n = 5)**
Increased waist circumstance	25 (48.1%)	12 (33.3%)	9 (75.0%)	4 (100.0%)	31 (62.0%)	18 (51.4%)	8 (80.0%)	5 (100.0%)
Decreased HDL	7 (13.5%)	0 (0.0%)	4 (33.3%)	3 (75.0%)	14 (28.0%)	5 (14.3%)	4 (40.0%)	5 (100.0%)
Hypertriglycerides	10 (19.2%)	2 (5.6%)	6 (50.0%)	2 (50.0%)	9 (18.0%)	2 (5.7%)	2 (20.0%)	5 (100.0%)
Hyperglycemia	19 (36.5%)	8 (22.2%)	7 (58.3%)	4 (100.0%)	12 (24.0%)	3 (8.6%)	6 (60.0%)	3 (60.0%)
High blood pressure	24 (46.2%)	10 (27.8%)	10 (83.3%)	4 (100.0%)	21 (42.0%)	8 (22.9%)	10 (100.0%)	3 (60.0%)

The data are expressed as a number (percentage). *MS*, metabolic syndrome, *HDL*, high density lipoprotein.

### Differential expression of circulating miRNAs between MetS and non-MetS subjects

**Figure 1:**
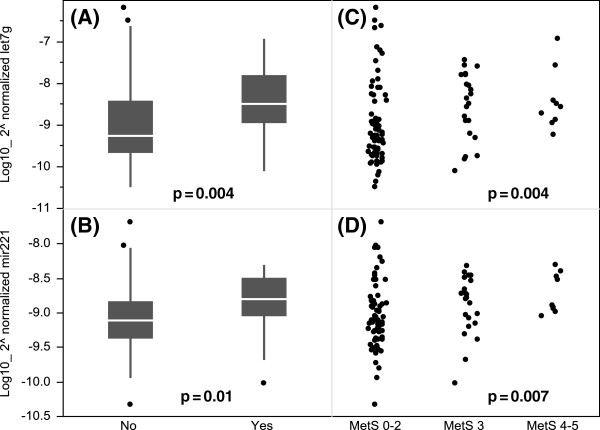
**Differential expression of serum miRNAs in individuals with or without MetS.** (Left: **A** and **B**) Comparisons were made between subjects without MetS (n = 71) and with MetS (n = 31) through non-parametric Mann–Whitney *U* tests. The box plots depict the 25th-75th percentile, and the white line represents the median of each contribution. (Right: **C** and **D**) The relationships between the number of MetS risk components and the levels of miRNA was analyzed through Jonckheere-Terpstra tests. The status of MetS was converted into an ordinal variable: MetS 0–2 (n = 71), MetS 3 (n = 22), and MS 4–5 (n = 9). Differences with p < 0.05 were statistically significant.

The comparisons between the MetS (n = 31) and the non-MetS subjects (n = 71) showed that the levels of both let-7 g and miR-221 were significantly elevated in the circulation of the MetS subjects (p = 0.004 for let-7 g and p = 0.01 for miR-221, Figure 1A and 1B). We further analyzed the relationship between the miRNAs levels and the number of MetS risk components. The levels of the two miRNAs were elevated with increasing numbers of MetS risk components: 0–2 components (n = 71), 3 components (n = 22), and 4–5 components (n = 9; p = 0.004 for let-7 g and p = 0.007 for miR-221, Figure 1C and 1D).

### Relationship between miRNAs and the MetS components

**Figure 2:**
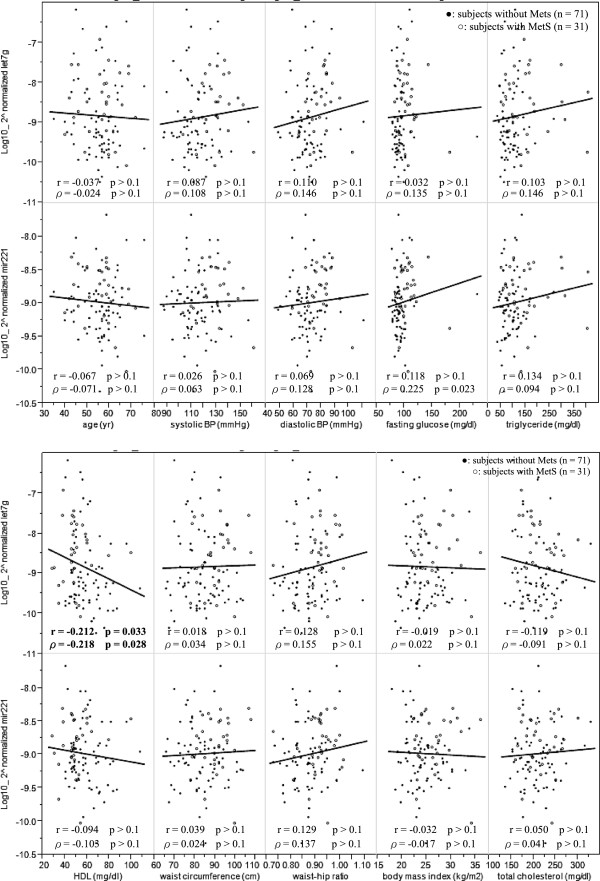
**The associations between the individual miRNAs and the MetS risk components.** Correlations between the expression level of the circulating miRNAs and the individual component of metabolic syndrome. The relationships were assessed by Pearson’s and Spearman’s correlation (r, Pearson’s correlation coefficient; ρ,Spearman’s coefficient). HDL, high-density lipoprotein cholesterol; BP, blood pressure.

We further tested the relationship between each miRNA and each individual MetS component (Figure 2). Of the five components of MetS and the two obesity parameters (body mass index and waist-hip ratio), the HDL cholesterol was the only factor that exhibited a significant correlation with the level of circulating let-7 g (r = -0.212, p = 0.033 and ρ = -0.218, p = 0.028 by Pearson’s and Spearman’s correlation, respectively). The finding suggests that plasma HDL cholesterol has an inverse relationship with the level of let-7 g in the circulation. The similar result was obtained after adjusting for age and smoking status (β = -0.017, 95% confidence interval = -0.031 ~ -0.004, p = 0.014 by multiple linear regression).

### Differential expression of circulating miRNAs between men and women

**Figure 3:**
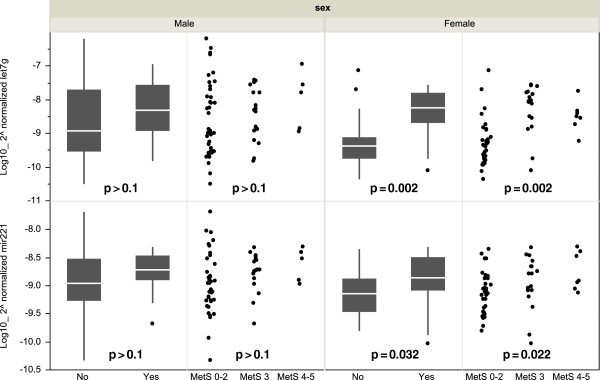
**Differential expression of serum miRNAs in male and female subjects with and without MetS.** The sample sizes of the male and female subjects were the following: MS(-) n = 36, MS(+) n = 16 in men and MS(-) n = 35, MS(+) n = 15 in women. (Left section) The comparisons between the individuals without or with MetS were conducted through non-parametric Mann–Whitney *U* tests. The box plots depict the 25th-75th percentile, and the white line represents the median of each contribution. (Right section) The relationships between the number of MetS risk components and the miRNA levels were analyzed by Jonckheere-Terpstra tests. The status of MetS was converted into an ordinal variable: MS score 0–2 (n = 36 in men and n = 35 in women), MS score 3 (n = 12 in men and n = 10 in women), and MS score 4–5 (n = 4 in men and n = 5 in women). Differences with p < 0.05 were statistically significant.

**Figure 4:**
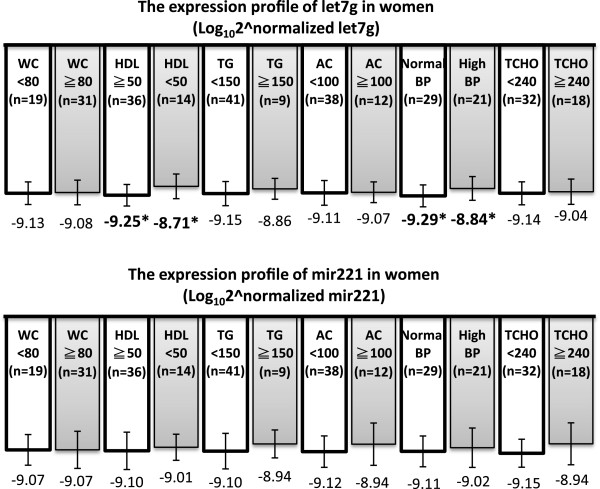
**Comparisons of the expression profiles of circulating miRNAs in women according to each abnormal components of MetS.** Female subjects were separated to two groups using the criteria of individual MetS components. * Significant difference (p < 0.05) between the groups adjusted by age. All of the data were analyzed through non-parametric Mann–Whitney *U* tests. WC, waist circumstance; HDL, high-density lipoprotein cholesterol; TG, triglycerides; AC, fasting blood sugar; BP, blood pressure; TCHO, total cholesterol.

The sex-specific analysis showed that the miRNA expression levels were distinct between men and women (Figure 3). Among the males (n = 52), the serum miRNA levels were not significantly different between the MetS (n = 16) and the non-MetS (n = 36) individuals (p > 0.1 for both miRNAs). In contrast, the two miRNAs were significantly overexpressed in the female subjects with MetS (n = 15) compared with those without MetS (n = 35; p = 0.002 for let-7 g; p = 0.032 for miR-221). Remarkably, the increased level of either miRNAs was paralleled with an increased number of MetS risk components. However, the statistical significance was only observed in the women subjects (p = 0.002 for let-7 g; p = 0.022 for miR-221). Among the females, each MetS component was defined as high or low using the abovementioned ATPIII definition. A higher level of circulating let-7 g was significantly associated with a higher blood pressure (p = 0.023) and a lower HDL cholesterol level (p = 0.022; Figure 4). The serum miR-221 level did not exhibit any correlation with any of the MetS risk components.

## Discussion

The present study revealed that two miRNAs (let-7 g and miR-221) are notably overexpressed in the serum of individuals with MetS. The sex-specific analysis implied that the two miRNAs are more significantly associated with MetS in women. The level of either miRNAs increased when the number of MetS risk components increased. Furthermore, let-7 g may also affect individual MetS risk components: an increase in the let-7 g level was correlated with a high blood pressure and a low HDL cholesterol level. However, miR-221 may have a less distinguishable effect on the individual risk components. Our data suggest that circulating let-7 g and miR-221 may be associated with the development of MetS.

Intracellular miRNAs can be secreted into an extracellular environment through two distinct mechanisms: intra-vesicular transport (exosomes, microparticles, and apoptotic bodies) and extra-vesicular transport (protein and lipoprotein complexes) [11]. Secreted extracellular miRNAs serve as signaling molecules and mediate intercellular crosstalk. The present available data have been demonstrated that microvesicle-enclosed and lipoprotein-delivered miRNAs can be transferred into recipient cells and further influence cell phenotypes and functions [25,26]. However, the exact mechanism of the release for an individual miRNA from different cell types is largely unknown.

Another implication of our data is that the elevated levels of circulating let-7 g and miR-221 in the MetS subjects may affect CV complications. Vascular endothelial cells (ECs), circulating endothelial progenitor cells (EPCs), and peripheral blood components can be in direct contact, and ECs and EPCs are exposed to circulating exosomes containing miRNAs that may influence the function of these cells and eventually contribute to cardiovascular diseases. The biological functions of miRNAs in vascular walls are cell-specific. Our recent publication demonstrated that let-7 g is involved in lipid-mediated atherosclerosis by negatively regulating an oxidized low-density lipoprotein receptor, LOX-1 [27]. Transfection of let-7 g into human aortic smooth muscle cell inhibited LOX-1 and the transcriptional factor OCT-1 expression, cell proliferation and migration. In addition, we also demonstrated that let-7 g could be downregulated in the aorta of mice fed a high-fat diet and that this result was accompanied by an increase in the expression of LOX-1. The present study demonstrated that an increase in the serum let-7 g level is associated with high blood pressure and low HDL cholesterol levels in women. Both high blood pressure and low HDL-cholesterol have been described in connection with LOX-1 overexpression in atherosclerotic lesions [28,29]. It is possible that circulating let-7 g can be taken up by vascular smooth muscle cells (VSMC) to inhibit LOX-1 expression. Therefore, we hypothesize that the elevated circulating let-7 g levels in MetS subjects can be a defense mechanism through which the body fights an unfavorable lipid profile. Conversely, the elevation of the circulating miR-221 levels may aggravate the process of atherosclerosis. A previous investigation showed that the level of miR-221 in EPCs was higher in patients with coronary artery disease and the miR-221 expression inversely correlated with the number of EPCs [30]. Zhang X et al. further demonstrated that miR-221 overexpression inhibited the proliferation of endothelial progenitor cells by directly targeting PAK1 and affecting the c-Raf/MEK/ERK pathway [31]. An *in vivo* study in rats also indicated that the downregulation of miR-221 and miR-222 can reduce the proliferation of VSMCs and neointimal hyperplasia, both of which play important roles in arterial restenosis after intima injury [32]. It is possible that the circulating miR-221 can be taken up by these cells and that this action might interfere with cell growth. However, further studies are necessary to confirm these findings.

Recent studies suggest that the contribution of individual metabolic syndrome component to relative risks of cardiovascular diseases and heart failure differs between genders [2,3,33]. A number of mechanisms have been considered for the gender disparity in MetS-associated cardiovascular sequelae, including sex hormones, neurovascular regulation, and intrinsic myocardial geometry and function [3]. One 12-year observational study demonstrated that the association between MetS and all-cause, cardiovascular, and cardiac mortality was only seen in postmenopausal women [4]. However, the reasons for the lack of significant relationship in men and premenopausal women were unclear. The current study indicated that the elevation of the circulating let-7 g and miR-221 levels was predominant in MetS women. Of interest, 80% of the female subjects with MetS were above the median age (50 years) of natural menopause in Chinese women. Although age and cigarette smoking are leading contributors to cardiovascular risks, in our study the expression of circulating let-7 g and miR-221 were not correlated with age, and none of women had the smoking habit. Beyond their roles in glucose metabolism, let-7 g and miR-221 play a prominent role in vascular smooth muscle cells and endothelial cells [34]. Our data provide a potential clue to explore the changes of vascular pathology in individuals with MetS, especially in women. Further investigations are required to understand how let-7 g and miR-221 affect MetS-related cardiovascular complications.

It is not uncommon to find sex-specific risk factors for common diseases. Similarly, miRNA studies have also reported a sex-specific pattern. A previous investigation demonstrated that the expression of miR-30b in the brain cortex of schizophrenic patients was only reduced in female subjects [35]. The study further showed that miR-30b can be regulated by estrogen signaling in neuronal tissues. In our study, there was no difference in the expression levels of let-7 g and miR-221 between women younger (n = 11) and older (n = 39) than the age of 50 years (-9.16 vs. -9.08 for let-7 g and -9.07 vs. -9.08 for miR-221; p > 0.1 for both miRNAs). Although the absence of evidence that supports a difference in the miRNA levels between the women in these two age groups, our current data implies that the menopausal status might not be a major confounder in the relationship between the two miRNAs and MetS. In addition, we observed a similar increased pattern of circulating miRNAs in the male subjects with MetS. However, the lack of statistical significance could be due to the small sample size and the larger subsample variation in the serum miRNA levels among men without MetS. In our study, the higher prevalences of hypertension (22.2%) and smoking status (27.8%) in the non-MetS men may be a confounder to the variances.

Another important finding in this study was that the percentage of hemolyzed samples was much higher in the subjects with MetS compared with those without MetS (35.4% vs. 4.1%, p < 0.0001). Hemolysis is a major factor for the false results that are obtained in routine blood biochemistry examinations. It is also known that hemolysis will cause a release of RBC-derived miRNAs into the serum [24]. Thus, even if the degree of hemolysis is low, the interference may be substantial if there is a low abundance of miRNAs in the serum. Hemolysis can take place *in vivo*, *in vitro*, or both. In the present study, the procedures used for sample collection, handling, and centrifugation were performed under the same protocols. Of the 20 samples that were discarded due to the presence of hemolysis over the cutoff value, 17 were collected from MetS subjects. Further analysis showed that the 20 discarded samples exhibited significantly lower (p = 0.0012) HDL cholesterol (46 mg/dl) compared with the analyzed samples (57 mg/dl). Similarly, the 20 samples also had a higher level of triglycerides (264 mg/dl vs. 116 mg/dl, p < 0.0001). Therefore, the possible reasons for the disproportionate amount of hemolysis in MetS subjects include difficult vascular access due to potential atherosclerosis or changes in the RBC membrane properties [36]. The change in the circulating levels of let-7 g and miR-221 might be the net result of many microvesicles secreted from distinct tissue cells. However, let-7 g is enriched in both reticulocytes and erythrocytes, whereas miR-221 is a reticulocyte-specific miRNA [37]. If hemolysis had not been excluded, we would have overestimated the strength of the association between these two miRNAs and the occurrence of MetS.

## Conclusions

Overall, we observed higher circulating levels of let-7 g and miR-221 in female subjects with MetS. However, this pattern was not detected in the male subjects. Further large-scale studies are needed to validate our findings.

## Abbreviations

MetS: Metabolic syndrome; CV: Cardiovascular; miRNAs: microRNAs; HG: High glucose; HDL: High-density lipoprotein; RBC: Red blood cells; VSMC: Vascular smooth muscle cells; ECs: Endothelial cells; EPCs: Endothelial progenitor cells; LOX-1: Lectin-like oxidized low-density lipoprotein receptor-1.

## Competing interests

The authors declare that they have no competing interests.

## Authors’ contributions

YTW performed experiments and PCT collected data. YTW carried out the final data analysis, created tables and figures, and wrote the manuscript. PCT and YCL contributed with valuable and critical discussion. CYH provided scientific input and participated in the preparation of the manuscript. SHHJ coordinated the study, provided content checking, and oversaw its performance. All authors read and approved the final manuscript.

## References

[B1] Lai MM Li CI Kardia SL Liu CS Lin WY Lee YD Chang PC Lin CC Li TC Sex difference in the association of metabolic syndrome with high sensitivity C-reactive protein in a Taiwanese population BMC Public Health 2010 10 429 10.1186/1471-2458-10-429 20663138 PMC2920887

[B2] Regitz-Zagrosek V Lehmkuhl E Weickert MO Gender differences in the metabolic syndrome and their role for cardiovascular disease Clin Res Cardiol 2006 95 136 147 10.1007/s00392-006-0351-5 16598526

[B3] Ren J Kelley RO Cardiac health in women with metabolic syndrome: clinical aspects and pathophysiology Obesity (Silver Spring) 2009 17 1114 1123 19214173 10.1038/oby.2009.8

[B4] Lin JW Caffrey JL Chang MH Lin YS Sex, menopause, metabolic syndrome, and all-cause and cause-specific mortality–cohort analysis from the Third National Health and Nutrition Examination Survey J Clin Endocrinol Metab 2010 95 4258 4267 10.1210/jc.2010-0332 20534759

[B5] Jakob P Landmesser U Role of microRNAs in stem/progenitor cells and cardiovascular repair Cardiovasc Res 2012 93 614 622 10.1093/cvr/cvr311 22135162

[B6] Jamaluddin MS Weakley SM Zhang L Kougias P Lin PH Yao Q Chen C miRNAs: roles and clinical applications in vascular disease Expert Rev Mol Diagn 2011 11 79 89 10.1586/erm.10.103 21171923 PMC3077058

[B7] Sayed D Abdellatif M MicroRNAs in development and disease Physiol Rev 2011 91 827 887 10.1152/physrev.00006.2010 21742789

[B8] Chen KC Hank Juo SH MicroRNAs in atherosclerosis Kaohsiung J Med Sci 2012 28 631 640 10.1016/j.kjms.2012.04.001 23217354 PMC11915937

[B9] Hunter MP Ismail N Zhang X Aguda BD Lee EJ Yu L Xiao T Schafer J Lee ML Schmittgen TD Detection of microRNA expression in human peripheral blood microvesicles PLoS One 2008 3 e3694 10.1371/journal.pone.0003694 19002258 PMC2577891

[B10] Kroh EM Parkin RK Mitchell PS Tewari M Analysis of circulating microRNA biomarkers in plasma and serum using quantitative reverse transcription-PCR (qRT-PCR) Methods 2010 50 298 301 10.1016/j.ymeth.2010.01.032 20146939 PMC4186708

[B11] Zampetaki A Willeit P Drozdov I Kiechl S Mayr M Profiling of circulating microRNAs: from single biomarkers to re-wired networks Cardiovasc Res 2012 93 555 562 10.1093/cvr/cvr266 22028337 PMC3291086

[B12] Karolina DS Tavintharan S Armugam A Sepramaniam S Pek SL Wong MT Lim SC Sum CF Jeyaseelan K Circulating miRNA profiles in patients with metabolic syndrome J Clin Endocrinol Metab 2012 97 E2271 2276 10.1210/jc.2012-1996 23032062

[B13] Hulsmans M De Keyzer D Holvoet P MicroRNAs regulating oxidative stress and inflammation in relation to obesity and atherosclerosis FASEB J 2011 25 2515 2527 10.1096/fj.11-181149 21507901

[B14] Heneghan HM Miller N McAnena OJ O’Brien T Kerin MJ Differential miRNA expression in omental adipose tissue and in the circulation of obese patients identifies novel metabolic biomarkers J Clin Endocrinol Metab 2011 96 E846 850 10.1210/jc.2010-2701 21367929

[B15] Frost RJ Olson EN Control of glucose homeostasis and insulin sensitivity by the Let-7 family of microRNAs Proc Natl Acad Sci USA 2011 108 21075 21080 10.1073/pnas.1118922109 22160727 PMC3248488

[B16] Zhu H Shyh-Chang N Segre AV Shinoda G Shah SP Einhorn WS Takeuchi A Engreitz JM Hagan JP Kharas MG The Lin28/let-7 axis regulates glucose metabolism Cell 2011 147 81 94 10.1016/j.cell.2011.08.033 21962509 PMC3353524

[B17] Li Y Song YH Li F Yang T Lu YW Geng YJ MicroRNA-221 regulates high glucose-induced endothelial dysfunction Biochem Biophys Res Commun 2009 381 81 83 10.1016/j.bbrc.2009.02.013 19351599 PMC2670889

[B18] Chartoumpekis DV Zaravinos A Ziros PG Iskrenova RP Psyrogiannis AI Kyriazopoulou VE Habeos IG Differential Expression of MicroRNAs in Adipose Tissue after Long-Term High-Fat Diet-Induced Obesity in Mice PLoS One 2012 7 e34872 10.1371/journal.pone.0034872 22496873 PMC3319598

[B19] Ortega FJ Moreno-Navarrete JM Pardo G Sabater M Hummel M Ferrer A Rodriguez-Hermosa JI Ruiz B Ricart W Peral B Fernandez-Real JM MiRNA expression profile of human subcutaneous adipose and during adipocyte differentiation PLoS One 2010 5 e9022 10.1371/journal.pone.0009022 20126310 PMC2814866

[B20] Xie H Lim B Lodish HF MicroRNAs induced during adipogenesis that accelerate fat cell development are downregulated in obesity Diabetes 2009 58 1050 1057 10.2337/db08-1299 19188425 PMC2671055

[B21] Li L Wu J Pu D Zhao Y Wan C Sun L Shen CE Sun W Yuan Z Shen Q Factors associated with the age of natural menopause and menopausal symptoms in Chinese women Maturitas 2012 73 354 360 10.1016/j.maturitas.2012.09.008 23026018

[B22] Chang C Chow SN Hu Y Age of menopause of Chinese women in Taiwan Suppl Int J Gynecol Obstet 1995 49 191 192 10.1016/0020-7292(95)02354-F 7649328

[B23] Henkelman S Rakhorst G Blanton J Van Oeveren W Standardization of incubation conditions for hemolysis testing of biomaterials Mater Sci Eng C 2009 29 1650 1654 10.1016/j.msec.2009.01.002

[B24] Kirschner MB Kao SC Edelman JJ Armstrong NJ Vallely MP Van Zandwijk N Reid G Haemolysis during sample preparation alters microRNA content of plasma PLoS One 2011 6 e24145 10.1371/journal.pone.0024145 21909417 PMC3164711

[B25] Chen X Liang H Zhang J Zen K Zhang CY Horizontal transfer of microRNAs: molecular mechanisms and clinical applications Protein Cell 2012 3 28 37 10.1007/s13238-012-2003-z 22314808 PMC4875218

[B26] Hulsmans M Holvoet P MicroRNA-containing microvesicles regulating inflammation in association with atherosclerotic disease Cardiovasc Res 2013 100 7 18 10.1093/cvr/cvt161 23774505

[B27] Chen KC Hsieh IC Hsi E Wang YS Dai CY Chou WW Juo SH Negative feedback regulation between microRNA let-7 g and the oxLDL receptor LOX-1 J Cell Sci 2011 124 4115 4124 10.1242/jcs.092767 22135361

[B28] Mehta JL Chen J Hermonat PL Romeo F Novelli G Lectin-like, oxidized low-density lipoprotein receptor-1 (LOX-1): a critical player in the development of atherosclerosis and related disorders Cardiovasc Res 2006 69 36 45 10.1016/j.cardiores.2005.09.006 16324688

[B29] Sawamura T Kakino A Fujita Y LOX-1: a multiligand receptor at the crossroads of response to danger signals Curr Opin Lipidol 2012 23 439 445 10.1097/MOL.0b013e32835688e4 22777292

[B30] Minami Y Satoh M Maesawa C Takahashi Y Tabuchi T Itoh T Nakamura M Effect of atorvastatin on microRNA 221 / 222 expression in endothelial progenitor cells obtained from patients with coronary artery disease Eur J Clin Invest 2009 39 359 367 10.1111/j.1365-2362.2009.02110.x 19371267

[B31] Zhang X Mao H Chen J-y Wen S Li D Ye M Lv Z Increased expression of microRNA-221 inhibits PAK1 in endothelial progenitor cells and impairs its function via c-Raf/MEK/ERK pathway Biochem Biophys Res Commun 2013 431 404 408 10.1016/j.bbrc.2012.12.157 23333386

[B32] Liu X Cheng Y Yang J Xu L Zhang C Cell-specific effects of miR-221/222 in vessels: molecular mechanism and therapeutic application J Mol Cell Cardiol 2012 52 245 255 10.1016/j.yjmcc.2011.11.008 22138289 PMC3664545

[B33] Regitz-Zagrosek V Lehmkuhl E Mahmoodzadeh S Gender aspects of the role of the metabolic syndrome as a risk factor for cardiovascular disease Gend Med 2007 4 S162 S177 18156101 10.1016/s1550-8579(07)80056-8

[B34] Zampetaki A Mayr M MicroRNAs in vascular and metabolic disease Circ Res 2012 110 508 522 10.1161/CIRCRESAHA.111.247445 22302757

[B35] Mellios N Galdzicka M Ginns E Baker SP Rogaev E Xu J Akbarian S Gender-Specific Reduction of Estrogen-Sensitive Small RNA, miR-30b, in Subjects With Schizophrenia Schizophr Bull 2012 38 433 443 10.1093/schbul/sbq091 20732949 PMC3329977

[B36] Anichkov DA Maksina AG Shostak NA Relationships between erythrocyte membrane properties and components of metabolic syndrome in women Med Sci Monit 2005 11 CR203 210 15795702

[B37] Chen SY Wang Y Telen MJ Chi JT The genomic analysis of erythrocyte microRNA expression in sickle cell diseases PLoS One 2008 3 e2360 10.1371/journal.pone.0002360 18523662 PMC2408759

